# Fluorescence microscopy for the diagnosis of smear-negative pulmonary tuberculosis in Ethiopia

**DOI:** 10.4102/ajlm.v9i1.810

**Published:** 2020-09-28

**Authors:** Gemeda Abebe, Dossegnaw Aragaw, Mulualem Tadesse

**Affiliations:** 1 School of Medical Laboratory Sciences, Faculty of Health Sciences, Jimma University, Jimma, Ethiopia; 2 Mycobacteriology Research Center, Jimma University, Jimma, Ethiopia

**Keywords:** sputum smear-negative, light-emitting diode fluorescent microscopy, bleach pretreatment, Ethiopia, health

## Abstract

**Background:**

Despite its low sensitivity, microscopy remains the main method for the diagnosis of pulmonary tuberculosis in most laboratories in Ethiopia. Few studies have evaluated the performance of light-emitting diode fluorescent microscopy (LED-FM) in bleach-concentrated smear-negative sputum specimens.

**Objective:**

This study aimed to evaluate the diagnostic performance of LED-FM for smear-negative pulmonary tuberculosis in Ethiopia.

**Methods:**

A total of 194 adult patients with a cough lasting for more than two weeks, and who had three direct smear-negative sputum tests for *Mycobacterium tuberculosis* by Ziehl-Neelsen light microscopy, were included. All direct Ziehl-Neelsen-stained smear-negative sputum samples were cultured and were also visualised by LED-FM. Smears for LED-FM were performed from bleach-concentrated sputum sediment. The diagnostic performance of the LED-FM was compared to the culture method (the reference standard).

**Results:**

Of the 194 smear-negative sputum specimens analysed, 28 (14.4%) were culture-positive and 21 (10.8%) were LED-FM-positive for *M. tuberculosis*. However, only 11 of the 21 (52.4%) LED-FM-positive patients had a confirmed tuberculosis diagnosis by culture. Light-emitting diode fluorescence microscopy (FM) had a sensitivity of 39.3% (95% confidence interval: 21.2–57.4) and specificity of 93.9% (95% confidence interval: 90.4–97.6). Ten LED-FM-positive specimens were culture-negative, and all of these specimens had scanty grading (1–19 bacilli per 40 fields on LED-FM).

**Conclusion:**

This study showed that implementation of LED-FM on bleach pre-treated and concentrated sputum can significantly improve the diagnosis of smear-negative pulmonary tuberculosis. However, all scanty grade, positive smears by LED-FM need to be confirmed by reference culture method.

## Introduction

Ethiopia is among the 30 highest-burden countries for tuberculosis, tuberculosis-HIV coinfection and multidrug-resistant tuberculosis in the world.^1^ According to the first national population-based tuberculosis survey report, the number of registered smear-negative tuberculosis cases exceeded that of smear-positive cases in 2010/11.^2^ This prevalence survey showed that smear-positive cases accounted for only 43% of culture-positive tuberculosis cases in Ethiopia.

Although sputum smear-positive cases are more infectious than smear-negatives, studies have reported that 17% of tuberculosis transmissions were due to sputum smear-negative but culture-positive cases.^3^ Moreover, smear-negative pulmonary tuberculosis cases are associated with longer health service delays due to delayed diagnosis. Such delays in diagnosis may delay initiation of treatment, and further tuberculosis transmission may occur.^4,5^

A major challenge in tuberculosis control is the lack of an accurate, cost-effective, widely available point-of-care test. The Cepheid Xpert® MTB/RIF assay (Cepheid, Sunnyvale, California, United States) is a molecular test with single-use cartridges on the GeneXpert Instrument System. The World Health Organization (WHO) has recommended Xpert MTB/RIF as the initial diagnostic test in individuals with presumptive multidrug-resistant or HIV-associated tuberculosis.^6^ The WHO has also recommended that Xpert MTB/RIF may be used as a follow on test to smear microscopy, especially in further testing of smear-negative specimens. Following the WHO recommendation, the Ethiopian Ministry of Health has started a rollout of the Xpert MTB/RIF assay in selected laboratories.^7^ Currently, few laboratories have access to Xpert MTB/RIF machines and smear microscopy remains the most widely used tuberculosis diagnostic tool in Ethiopia.^7,8^ However, Ziehl-Neelsen (ZN) smear microscopy was found to have low sensitivity, ranging from 20% to 50%.^8,9,10,11^ On the other hand, fluorescence microscopy (FM), both conventional and light-emitting diode (LED) using Auramine O, has showed increased detection of tuberculosis bacilli by 10%.^12,13^ However, conventional FM use has been limited by the short life span of mercury vapour light, the need for regular maintenance and the requirement for a dark room.^14^ As a result, in 2011, the WHO recommended the replacement of conventional FM by light-emitting diode fluorescent microscopy (LED-FM) as an alternative for ZN light microscopy.^15^

LED-FM increases the sensitivity of smear microscopy because of the fact that slides can be examined at a lower magnification, allowing the examination of a much larger area per unit of time.^12,13,16^ This is particularly important in high tuberculosis burden and workload countries such as Ethiopia, where technicians spend much of their time on examining smears. There is limited data on the diagnostic performance of LED-FM in bleach-treated and concentrated sputum specimens, particularly paucibacillary samples. In this study, we aimed to evaluate the diagnostic performance of LED-FM in bleach pre-treated and concentrated sputum specimens for the diagnosis of smear-negative pulmonary tuberculosis in Ethiopia.

## Methods

### Ethical considerations

This study was reviewed and approved by the institutional review board of Jimma University (Ref. No: RPGC/510/2014). Consent was obtained from all participants for use of routine clinical data for research purposes. Laboratory results were reported back to the clinicians for treatment initiation or decision as early as available.

### Study setting and participants

This cross-sectional study was conducted at the Jimma University Specialized Hospital, a teaching and referral hospital, in Southwest Ethiopia from February 2014 to August 2014. Adult patients (age > 18 years) presenting with a cough (≥ 2 weeks) and who had had three negative tests for *Mycobacterium tuberculosis* by ZN sputum direct smear microscopy, were included in the study. Patients taking anti-tuberculosis treatment or prophylaxis in the four weeks before the screening were excluded.

### Study procedures

Adult presumptive pulmonary tuberculosis cases were requested to submit sputum specimens. Inclusion criteria was based on the WHO case definition for a pulmonary tuberculosis suspect^17^ and included patients having a persistent cough (≥ 2 weeks), with or without one of the following symptoms: night sweats, unintentional weight loss, fever, chest pain, shortness of breath, loss of appetite, and contact with a tuberculosis patient. As part of routine practice, all patients gave three sputum samples (spot-morning-spot). Sputum samples were examined by ZN sputum smear microscopy. Smears were considered positive if acid-fast bacilli (AFB) were seen on the smear from any of the three sputum samples. Sputum smear-positive patients were treated for tuberculosis according to the National Tuberculosis and Leprosy programme treatment guidelines. For the current analysis, patients who had three smear-AFB-negative sputum tests by ZN microscopy were included in the study. Demographic and clinical characteristics were collected through interview by use of a questionnaire.

### Smear microscopy by Ziehl-Neelsen

Sputum smear examination by the ZN staining technique is the standard method for the diagnosis of pulmonary tuberculosis in Ethiopia.^8^ A portion of patient sputum specimen was used for direct smear microscopy using the ZN method and the remaining portion was stored at 2 °C – 8 °C in a refrigerator for the culture and LED-FM tests.

For the ZN method, a direct smear was prepared on the spot during specimen collection on a clean slide. Then standard ZN staining procedure was applied. The stained smears were examined for AFB under the oil immersion objective lens in a light microscope (Olympus CX31 light microscope; Olympus, Tokyo, Japan). Smear results were reported for the presence or absence of AFB using the World Health Organization/International Union Against Tuberculosis and Lung Disease scale, with a positive result corresponding to ≥ 1 AFB per 100 high-power fields.^18^

### Bleach processing and concentration of sputum

The morning sputum sample was divided into two equal parts. The first half was transferred to a 15 mL conical centrifuge tube (SARSTEDT AG & Co. KG, Nümbrecht, Germany) and treated with an equal volume of 5% bleach (Chora Gas and Chemical Products Factory, Addis Ababa, Ethiopia). After mixing, the tubes were left for 15 minutes at room temperature with shaking for 30 seconds every 5 min. Then phosphate buffered saline was added up to 15 mL and centrifuged at 3000 revolutions per minute for 15 min using a simple centrifuge. This is a small, compact, bench-top, non-refrigerated centrifuge that is easy and safe for operation and is available in the peripheral laboratories of Ethiopia. After centrifugation, the supernatant was decanted, the sediment was re-suspended with 1 mL of phosphate buffered saline (pH = 6.8), then the smear was prepared for LED-FM.

### Light-emitting diode fluorescent microscopy

Smears were prepared on frosted slides and completely covered with Auramine O solution (Sigma-Aldrich, Machelen, Belgium). After 20 min, the slides were washed and decolourised with 0.5% acid alcohol solution for 3 min and counter-stained with 0.5% potassium permanganate for 1 min. Stained smears were examined under LED-FM (Primo Star iLED, Carl Zeiss, Gottingen, Germany) with 400X magnification and 40 fields were examined. LED-FM results were reported for the presence or absence of AFB using the World Health Organization/International Union Against Tuberculosis and Lung Disease scale, with a positive result corresponding to ≥ 1 AFB per 20x for screening and 40x for confirmation.^19^

### Sputum culture and identification

Culture was done both on Lowenstein–Jensen (LJ) slants and Mycobacteria growth indicator tubes (MGIT). Briefly, the second half of the morning sputum sample was decontaminated using the *N*-acetyl-L-cysteine and sodium hydroxide (NALC/NaOH) method with a final NaOH concentration of 1%.^20^ An equal volume of standard NALC/NaOH solution was added to the specimen and incubated for 15 min. After centrifugation for 15 min at 3000 x g, the sediment was re-suspended in 1 mL of sterile phosphate buffered saline. A total of 500 *µ*L of the resulting pellets was inoculated into the MGIT and 100 *µ*L onto the LJ tubes. In each run, *M. tuberculosis* strain H_37_Rv was used as a positive control. For the negative control, random LJ slants and MGIT tubes were inoculated with sterile phosphate buffered saline. Differentiation of the *M. tuberculosis* complex from non-tuberculous mycobacteria was performed using the SD BIO LINE MPT64 TB Ag test (Standard Diagnostics, Yongin, South Korea).

### Statistical analysis

Data were analysed using IBM SPSS Statistics for Windows, Version 20.0. (2011; IBM Corp., Armonk, New York, United States). Descriptive statistics were used for analysis of demographic and clinical characteristics. Sensitivity, specificity, and predictive values of the LED-FM, and respective 95% confidence interval (95% CI), were calculated with culture method as reference standard. *Mycobacterium tuberculosis* culture positivity was defined as identification of *M. tuberculosis* on the LJ medium and/or MGIT culture. We excluded data of patients with contaminated culture results from the diagnostic accuracy analysis.

## Results

### Characteristics of study participants

**FIGURE 1:**
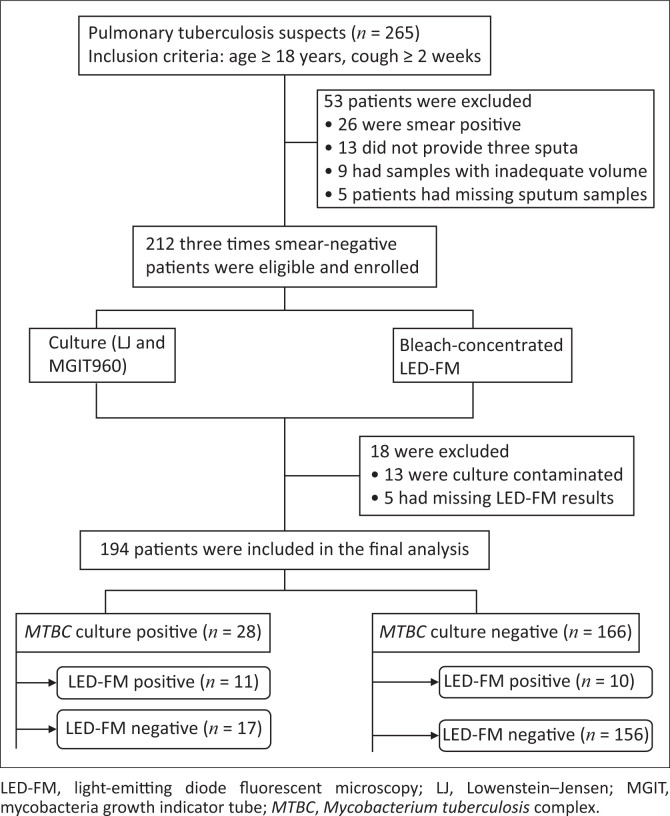
Flow diagram of patient recruitment, sample processing and diagnostic test results, Ethiopia, 2014.

A total of 265 consecutive adult patients with suggestive symptoms of pulmonary tuberculosis were screened. Fifty-three patients were excluded from the study (26 were sputum AFB smear-positive by ZN microscopy, 13 did not provide three sputum samples, 9 had samples with inadequate volume and 5 patients had missing sputum samples). Of the remaining 212 patients, 18 were excluded because of contaminated culture or missing LED-FM results, leaving 194 patients for the final analysis (Figure 1).

Of the 194 presumptive cases, 60.8% (118/194) were male. The median age of patients was 38 years (interquartile range 23–55). The majority (57.2%, 111/194) of patients had received antibiotics from local clinicians before the patients were referred to our hospital. Thirty-nine (20%) patients recalled that they had previous contact with known tuberculosis patients. All patients were currently not taking anti-tuberculosis treatment but 26.3% (51/194) of the cases had a history of previous anti-tuberculosis treatment (Table 1).

**TABLE 1:** Demographic and clinical characteristics of the study participants (*n* = 194), Ethiopia, 2014.

Characteristics	Smear-negative pulmonary tuberculosis suspects						*p*-value
	All patients (*n* = 194)		Culture-positive (*n* = 28)		Culture-negative (*n* = 166)		
	*n*	%	*n*	%	*n*	%	
Age, median (IQR)	38	23–55	32	21–47	40	26–56	0.27
Gender, male	118	60.8	20	71.4	98	59.0	0.21
Taking antibiotics in last 2 weeks	111	57.2	13	46.4	98	59.0	0.21
Contact history with tuberculosis patient	39	20.1	8	28.6	31	18.7	0.23
History of anti-TB treatment	51	26.3	5	17.9	46	27.7	0.27
**Presenting tuberculosis symptoms**							
Cough > 4 weeks	98	50.5	16	57.1	82	49.4	0.41
Chest pain	124	63.9	18	64.3	106	63.9	0.96
Night sweats	137	70.6	22	78.6	115	69.3	0.32
Fever (> 38.5 °C)	129	66.5	20	71.4	109	65.7	0.55
Weight loss	115	59.3	19	67.9	96	57.8	0.31
Shortness of breath	127	66.5	16	57.1	111	66.9	0.32
Loss of appetite	135	69.6	22	78.6	113	68.1	0.26
Weakness	158	81.4	23	82.1	135	81.3	0.91
Haemoptysis	26	13.4	6	21.4	20	12.0	0.18

TB, tuberculosis; IQR, interquartile range.

All patients included in this study had a cough for at least two weeks. About half (50.5%, 98/194) of the patients were coughing for more than four weeks before admission to our hospital. The most common clinical symptoms observed in these patients were weakness in 81.4% (158/194), night sweats in 70.6% (137/194), loss of appetite in 69.6% (135/194), fever in 66.5% (129/194), and weight loss in 59.3% (115/194). Haemoptysis was observed less frequently (13.4%, 26/194) (Table 1). We used an unadjusted logistic regression model to determine the predictors of smear-negative, culture-positive cases. None of the predictors analysed were associated with smear-negative pulmonary tuberculosis (*p*-value > 0.05).

### Accuracy of light-emitting diode fluorescent microscopy

Sputum was culture positive for *M. tuberculosis* in 14.4% (28/194) of ZN smear-negative patients. Twenty-one of the 194 patients with negative sputum (ZN method) had positive sputum smear examinations by LED-FM from bleach-concentrated sputum, but only 11 of these 21 patients (52.4%) had a confirmed tuberculosis diagnosis by culture (LJ and/or MGIT).

Among patients with negative sputum culture, LED-FM gave positive results in 10 cases; however, these had scanty grading. Patients’ cards for all LED-FM-positive, culture-negative patients were retrospectively reviewed to evaluate their treatment outcome. Only five of these patients started anti-tuberculosis treatment based on clinical criteria and three of them responded positively to anti-tuberculosis treatments. Two LED-FM positive patients did not respond to anti-tuberculosis treatment. For the remaining five, treatment outcome information was not available.

LED-FM gave positive results in 11 of 28 culture-positive samples and in 10 of 166 culture negatives. Using culture as the reference standard, the sensitivity of LED-FM for diagnosing smear-negative pulmonary tuberculosis was 39.3% (95% CI: 21.2–57.4) and the specificity was 93.9% (95% CI: 90.4–97.6). The positive predictive value for LED-FM was 52.4% (95% CI: 31–73.7), and the negative predictive value was 90.2% (95% CI: 85.7–94.6) (Table 2).

**TABLE 2:** Diagnostic performance of light-emitting diode fluorescence microscopy compared to reference standard (culture), Ethiopia, 2014.

Diagnostic accuracy of LED-FM	Value	95% confidence interval
Sensitivity	39.3%	21.2% – 57.4%
Specificity	93.9%	90.4% – 97.6%
Positive predictive value	52.4%	31.0% – 73.7%
Negative predictive value	90.2%	85.7% – 94.6%
Likelihood ratio positive	6.5	3.0 – 13.9
Likelihood ratio negative	0.65	0.48 – 0.87
Pre-test probability	14.4%	-
Positive post-test probability	52.4%	-

LED-FM, light-emitting diode fluorescence microscopy.

## Discussion

In this study, we reported a significant increase in detection of smear-negative pulmonary tuberculosis by using LED-FM on bleach-concentrated sputum specimens. Rapid identification and treatment of tuberculosis cases is the keystone of tuberculosis control. Although Xpert MTB/RIF is the diagnostic test of choice for tuberculosis, only a few laboratories in Ethiopia have access to the Xpert MTB/RIF assay. Moreover, in laboratories where Xpert MTB/RIF is available, there is a continuous stock-out of cartridges. Thus, in most laboratories, case detection relies primarily on identification of AFB in non-concentrated sputum ZN smears using a conventional light microscope.^8^ Conventional microscopy is inexpensive, rapid and highly specific, but has poor sensitivity, resulting in a high rate of missed cases.^12,21,22,23,24^

Light-emitting diode-based FM has been proposed as a technique to increase the sensitivity of smear examination. Previous studies showed that LED-FM was approximately 10% more sensitive than conventional microscopy using ZN and had comparable specificity.^13,25^ Few studies have evaluated the performance of LED-FM using bleach-concentrated sputum samples for the diagnosis of smear-negative pulmonary tuberculosis, and none of them were from Ethiopia.^14,25^ Bleach, or sodium hypochlorite, is cheap and available almost anywhere as household bleach. As a potent disinfectant, bleach also has the advantage of limiting the risk of laboratory infections. In addition, the relative centrifugal force needed for the concentration of mycobacteria was lower after digestion with bleach and can easily be achieved by a low-cost, table-top centrifuge that can easily be used under existing conditions in tuberculosis laboratories in developing countries such as Ethiopia.

In the current study, LED-FM yielded a positive result in a significant proportion of AFB sputum smear-negative patients. Light-emitting diode FM detected tuberculosis bacilli in 39% of culture-positive but ZN smear-negative patients. Light-emitting diode FM was found to be more sensitive than ZN smear microscopy. Similar results have been reported by different investigators.^16,24^ Possible explanations for increased sensitivity of LED-FM may be because of a stronger affinity of Auramine than carbol-fuchsin to mycolic acid. With LED-FM, slides can be examined at a lower magnification, thus allowing quick examination, which would favour increased sensitivity. Another reason for increased sensitivity of LED-FM could be related to the fact that LED-FM was performed after bleach processing and centrifugation. Bleach processing has been reported to improve the identification of bacilli by providing a clearer microscopy field through digestion of mucus and concentrating bacilli by means of centrifugation. Had results of LED-FM been made available to the clinicians, 11 (39.3%) additional patients could correctly have been started on anti-tuberculosis treatment.

Even though we observed increased sensitivity of LED-FM in bleach-processed and concentrated sputum, there were 17 culture-positive cases that were negative by LED-FM in this study. The low number of tuberculosis bacilli present in smear-negative sputum could be the reason for false-negative LED-FM test results. A negative result with LED-FM does not rule out a diagnosis of smear-negative tuberculosis, given the fact that LED-FM was unable to identify 60.7% of patients with culture-confirmed pulmonary tuberculosis in this study. Therefore, patients with a strong clinical suspicion of pulmonary tuberculosis despite a negative LED-FM should be initiated on anti-tuberculosis treatment. Despite this, LED-FM can provide a faster turn-around time, minimising loss to follow up during diagnostic evaluation of smear-negative pulmonary tuberculosis patients.

In our study, 10 LED-FM positive specimens were culture-negative. All of these smears had scanty grading. Slightly lower or comparable specificity of LED-FM compared with conventional ZN microscopy has been reported in previous studies.^16,25^ On certain occasions, Auramine O can stain cellular debris or other artefacts and may lead to false positive LED-FM results. On the other hand, negative culture results of these 10 cases may be due to over-decontamination of specimens. Specimens with low numbers of tuberculosis bacilli are particularly prone to being over-decontaminated and can result in false-negative cultures.^26^ It is of paramount importance to ascertain that culture-negative LED-FM-positive cases are unambiguously true tuberculosis cases. Therefore, we recommend further clinical evaluation study which specifically addresses such cases.

### Limitations

This study is not without limitations. Light-emitting diode FM was performed only on bleach pre-treated and concentrated sputum samples. Ideally, LED-FM should be done on both bleach pre-treated and concentrated samples as well as non-bleach pre-treated and concentrated samples. Then the effect of bleach pretreatment and concentration in the diagnosis of smear-negative tuberculosis will be evaluated. However, this was not done in the current study.

### Conclusion

We concluded that, of those patients found to have a negative result in three consecutive sputum examinations with ZN, a significant proportion (10.8%) had a positive result using LED-FM after bleach processing and centrifugation. Light-emitting diode FM improves the speed and increases the detection rate of smear-negative pulmonary tuberculosis, although all scanty grades need to be confirmed with the standard method. In resource-limited settings where there is no Xpert MTB/RIF and culture, LED-FM in bleach-processed and concentrated sputum can be considered complementary to conventional ZN smear microscopy. Future studies on the overall yield of LED-FM in bleach-processed sputum with a larger sample size are warranted for routine diagnosis of patients suspected of having smear-negative pulmonary tuberculosis in resource-limited countries.
